# Real-world PD-L1 testing and distribution of PD-L1 tumor expression by immunohistochemistry assay type among patients with metastatic non-small cell lung cancer in the United States

**DOI:** 10.1371/journal.pone.0206370

**Published:** 2018-11-08

**Authors:** Vamsidhar Velcheti, Pallavi D. Patwardhan, Frank Xiaoqing Liu, Xin Chen, Xiting Cao, Thomas Burke

**Affiliations:** 1 Hematology and Oncology, New York University, Perlmutter Cancer Center, New York, New York, United States of America; 2 Center for Observational and Real-world Evidence (CORE), Merck & Co., Inc., Kenilworth, New Jersey, United States of America; University of South Alabama Mitchell Cancer Institute, UNITED STATES

## Abstract

**Background:**

The anti-programmed death receptor-1 (anti–PD-1) pembrolizumab is approved as first-line monotherapy for metastatic non-small cell lung cancer (mNSCLC) with PD-ligand 1 (PD-L1) tumor expression ≥50%. Most studies comparing PD-L1 results by immunohistochemistry (IHC) assay type have been conducted by prespecified and, in most cases, highly experienced, trained pathologists; however, knowledge is limited regarding the current use and concordance of PD-L1 assays in the real-world clinical setting. Our aim was to study the distribution of PD-L1 tumor expression by IHC assay type among patients with mNSCLC in US oncology practices.

**Methods:**

This retrospective observational study utilized de-identified, longitudinal data from a large US electronic medical record database. Eligible patients were adults (≥18 years) with histologically/cytologically confirmed initial diagnosis of metastatic or recurrent NSCLC from October 2015 through December 2017. We determined PD-L1 testing trends and distribution of PD-L1 tumor expression (percentage of tumor cells staining for PD-L1) by IHC assay type.

**Results:**

The 12,574 eligible patients (mean age, 69 years) included 6,620 (53%) men and 86% with positive smoking history. Of 4,868 evaluable tests, 3,799 (78%), 195 (4%), 165 (3%), and 709 (15%) used the Agilent 22C3 pharmDx, Agilent 28–8 pharmDx, Ventana PD-L1 (SP142) Assay, and laboratory-developed tests (LDTs, including SP263), respectively. The percentages of tests scoring PD-L1 tumor expression of ≥50% were 33%, 32%, 10%, and 23%, respectively. Measured PD-L1 tumor expression varied across the four assay types (χ^2^ p < 0.001) and across three assay types excluding SP142 (p < 0.001), with no significant difference between 22C3 and 28–8 assays (p = 0.96). The PD-L1 testing rate increased from 18% in the fourth quarter of 2015 to 71% in the fourth quarter of 2017.

**Conclusions:**

In the real-world clinical setting, we observed that measured PD-L1 tumor expression is concordant using the 22C3 and 28–8 assays; however, the SP142 assay and LDTs appear discordant and could underestimate high PD-L1 positivity. Further study is needed to evaluate the association between PD-L1 tumor expression and response to therapy.

## Introduction

The introduction of anti-programmed death receptor-1 (anti-PD-1) antibodies, pembrolizumab and nivolumab, and the anti-programmed death-ligand 1 (anti-PD-L1) antibodies, such as atezolizumab and durvalumab, has dramatically changed the treatment landscape for non-small cell lung cancer (NSCLC). Currently in the US, pembrolizumab is the only one of these agents approved by the US Food and Drug Administration (FDA) for first-line monotherapy of metastatic NSCLC (for tumors negative for epidermal growth factor receptor/anaplastic lymphoma kinase [*EGFR/ALK*] genomic alterations). In the second line setting, pembrolizumab, nivolumab, and atezolizumab are all approved for advanced and metastatic NSCLC therapy upon disease progression after platinum-based chemotherapy and treatment for *EGFR/ALK* genomic alterations, if indicated.

Pembrolizumab therapy is approved in conjunction with a companion diagnostic, the PD-L1 IHC 22C3 pharmDx test (Agilent Technologies, Carpinteria, CA), for identifying those patients who are likely to benefit from pembrolizumab [1–5]. As first-line therapy, pembrolizumab is approved as monotherapy for metastatic NSCLC with high tumor expression of PD-L1 (tumor proportion score [TPS] of ≥50%), and in combination with pemetrexed and carboplatin for metastatic NSCLC with nonsquamous histology regardless of PD-L1 expression level. In addition, pembrolizumab is approved as second-line monotherapy for advanced or metastatic NSCLC with PD-L1 TPS ≥1%. However, testing for PD-L1 tumor expression is not a labeled requirement for use of nivolumab and atezolizumab, although FDA-approved drug-specific complementary PD-L1 diagnostic assays were developed for use in clinical trials, namely, the Agilent PD-L1 IHC 28–8 pharmDx for nivolumab and the Ventana PD-L1 (SP142) Assay (Ventana Medical Systems, Tucson, AZ) for atezolizumab [6–9]. Another available PD-L1 assay is the Ventana PD-L1 (SP263) Assay, developed for use with durvalumab, a PD-L1 antibody FDA-approved for treating urethelial carcinoma and unresectable Stage III NSCLC. In addition, some centers are using laboratory-developed tests (LDTs) for PD-L1 that employ various combinations of primary antibodies and IHC autostainer platforms [6,10,11].

The utilization of different PD-L1 IHC assays, as well as the different PD-L1 tumor expression cut-points used in clinical trials, raised concerns in the oncology community as early as 2015 regarding the concordance among these assays and the optimal approach for measuring PD-L1 [12–14]. The Blueprint PD-L1 IHC Assay Comparison Project is an industrial-academic partnership seeking to harmonize IHC PD-L1 testing [10]. Results of Blueprint phase 1, a feasibility study evaluating the analytical comparability of four assays (22C3, 28–8, SP263, and SP142) assessed independently by three experts, indicated that PD-L1 tumor expression (cell staining) was concordant for the 22C3, 28–8, and SP263 assays, while the SP142 assay consistently showed fewer stained tumor cells [10]. The Blueprint study phase 2 confirmed the findings of phase 1 using real-world routine clinical samples from 81 patients, with concordant results as read by 25 trained pathologists [15]. Several other validation studies have reported similar findings [6,11,16–19].

Recent evidence suggests, however, that, while the 22C3 and 28–8 assays may be interchangeable, potential differences can occur among laboratories and individual pathologists in applying specific PD-L1 cut-points, particularly when using LDTs that are not properly validated [11,18] and particularly at lower cut-points [16,17]. The need to standardize PD-L1 testing is highly relevant in the clinical setting, where clinicians must choose the optimal treatment regimen for their patients with NSCLC. Evidence from a recent meta-analysis indicates improved overall response rates with anti-PD-1/anti-PD-L1 therapy in NSCLC when used to treat chemotherapy-naïve patients as compared with patients previously treated with chemotherapy [20], highlighting the importance of identifying patients who will benefit from first-line anti-PD-1/anti-PD-L1 therapy.

Prior studies comparing PD-L1 results by IHC assay type have been conducted by prespecified and, in most cases, highly experienced, trained pathologists. However, knowledge is limited regarding the current use and concordance of PD-L1 assays in the real-world clinical setting. The objectives of this retrospective study were to study the distribution of PD-L1 tumor expression by assay type among patients with metastatic NSCLC treated at US oncology practices and to assess recent PD-L1 testing patterns.

## Methods

### Data source

This was a retrospective cohort study utilizing longitudinal electronic health record data from the Flatiron Health database [21]. At the time of our study, this database included de-identified data from active records of more than 2 million patients with cancer in the United States. Both structured and unstructured patient-level data are included in the Flatiron database, which is refreshed monthly. The structured data include laboratory values, limited biomarker information, and prescribed drugs, while unstructured data include information taken from unstructured documents, such as physician’s notes in the medical record and detailed biomarker, radiology, and pathology reports.

### Study design and patient population

The population for this study was derived from patients with advanced NSCLC represented in the Flatiron Health database, which has the following inclusion criteria: (1) diagnosis of advanced NSCLC, confirmed via review of unstructured documentation, and (2) at least two clinical visits documented in the Flatiron dataset on or after January 1, 2011.

Patients who received an initial diagnosis of metastatic or recurrent NSCLC from October 2, 2015 (the first US FDA approval date for pembrolizumab in second-line treatment of metastatic NSCLC with PD-L1 TPS ≥50% [22]), through December 31, 2017 (most recent data cut from Flatiron Health at the time of this analysis), were included in the study. Eligible patients were 18 years or older at the time of diagnosis, which was histologically or cytologically confirmed. Patients were excluded if they had a PD-L1 tumor expression test date recorded before October 2, 2015.

This was a non-interventional study using anonymized data; therefore, informed consent was not possible or necessary. No patient identifying information was accessible during the study, and the data in the Flatiron Health database are protected against breach of confidentiality [21].

### Study outcome measures

We evaluated PD-L1 testing information for all eligible patients, including the IHC assay type and the distribution of PD-L1 tumor expression by IHC assay type. The assays included were three FDA-approved IHC assays (Agilent 22C3 pharmDx, Agilent 28–8 pharmDx, Ventana PD-L1 SP142 assay) and all LDTs pooled (including Ventana SP263). We defined the test date as the results date, and PD-L1 tumor expression as the percentage of tumor cells staining for PD-L1. At the time of the study, the database did not capture results for immune cell staining seen with the SP142 assay. Patients could have more than one PD-L1 test and/or assay type.

### Statistical analyses

We used descriptive statistics to summarize patient demographic and clinical characteristics at baseline and the percentage of patients tested, by PD-L1 assay type. For patient baseline characteristics, continuous and count variables were compared using a *t* test, and binary and categorical variables were compared using the χ^2^ test.

The χ^2^ test was used to compare PD-L1 tumor expression, categorized as <1%, 1–49%, and ≥50%, (1) across four assay types including the SP142, (2) across three assay types (22C3, 28–8, and LDTs, which also included the SP263) excluding the SP142, and (3) between two assay types (22C3 and 28–8).

Statistical analyses were performed using SAS 9.4 software (SAS Institute Inc., Cary, NC).

## Results

### Patients

**Fig 1:**
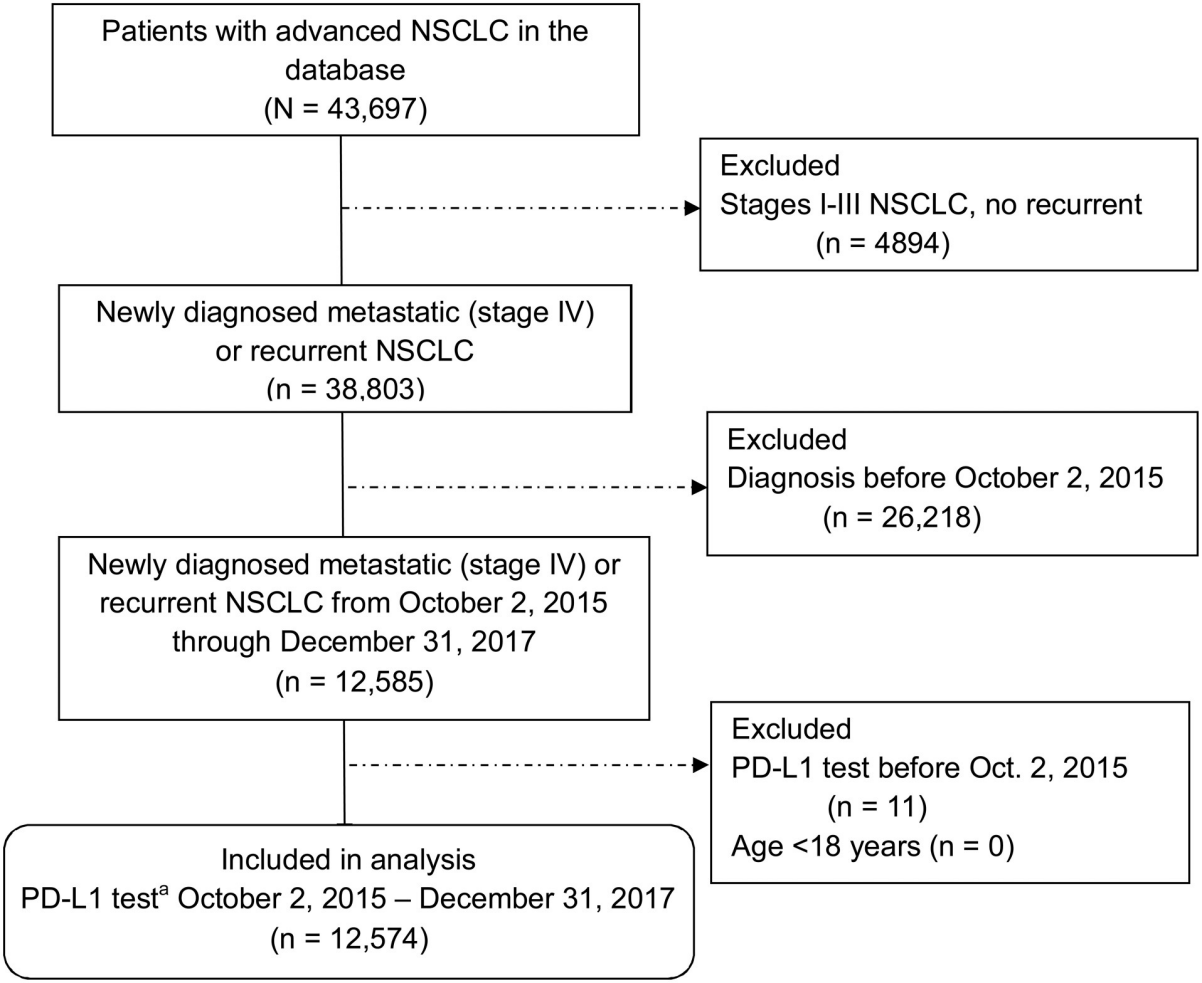
Selection of eligible patients with metastatic or recurrent non-small cell lung cancer from the database. NSCLC, non–small-cell lung cancer; PD-L1, programmed death-ligand 1. ^a^All testing for PD-L1 was required to be on or after October 2, 2015; however, patients were not required to have a PD-L1 test to be eligible for the study.

Of 12,585 patients with metastatic or recurrent NSCLC diagnosis on or after October 2, 2015, through December 31, 2017, we identified 12,574 (>99%) patients eligible for the study (Fig 1), ranging in age from 20–85 years (mean 69 years) and including 6620 (53%) men (Table 1). Overall, 9048 (72%) patients had nonsquamous NSCLC, 2821 (22%) had squamous NSCLC, and 705 (6%) had NSCLC not otherwise specified (NOS).

**Table 1:** Baseline demographic and clinical characteristics of patients with metastatic or recurrent NSCLC by PD-L1 testing status.

Characteristic	All patients (N = 12,574)	Tested for PD-L1 (n = 6024)	Not tested for PD-L1 (n = 6550)	p Value^a^
Age at metastatic diagnosis, years				
Mean (SD)	69.2 (10.0)	68.8 (10.3)	69.5 (9.8)	<0.001
≤65 years	4317 (34.3)	2163 (35.9)	2154 (32.9)	<0.001
>65 years	8257 (65.7)	3861 (64.1)	4396 (67.1)	
Male sex	6620 (52.6)	3127 (51.9)	3493 (53.3)	0.11
Race, data available^b^	10,945 (87.0)	5238 (87.0)	5707 (87.1)	
White	8364 (76.4)	3952 (75.4)	4412 (77.3)	0.15
Black	1035 (9.5)	509 (9.7)	526 (9.2)	
Asian	357 (3.3)	183 (3.5)	174 (3.0)	
Other	1189 (10.9)	594 (11.3)	595 (10.4)	
Smoking history, data available^b^	12,484 (99.3)	5993 (99.5)	6491 (99.1)	
Positive smoking history	10,778 (86.3)	5105 (85.2)	5673 (87.4)	<0.001
Stage available at first diagnosis^b^	12,123 (96.4)	5868 (97.4)	6255 (95.5)	
Stage II or lower	1768 (14.6)	679 (11.6)	1089 (17.4)	<0.001
Stage III/IIIA	1133 (9.3)	349 (5.9)	784 (12.5)	
Stage IV	9222 (76.1)	4840 (82.5)	4382 (70.1)	
Histology				
Nonsquamous cell carcinoma	9048 (72.0)	4,596 (76.3)	4452 (68.0)	<0.001
Squamous cell carcinoma	2821 (22.4)	1144 (19.0)	1677 (25.6)	
NSCLC NOS	705 (5.6)	284 (4.7)	421 (6.4)	
Tested for EGFR/ALK	8870 (70.5)	5214 (86.6)	3656 (55.8)	<0.001
Practice type				
Academic	1159 (9.2)	555 (9.2)	604 (9.2)	0.99
Community	11,415 (90.8)	5469 (90.8)	5946 (90.8)	

Data presented as No. (%) unless otherwise noted. Percentages may not add up to 100% because of rounding. ALK, anaplastic lymphoma kinase rearrangement; EGFR, epidermal growth factor receptor mutation; NSCLC NOS, non-small cell lung cancer, not otherwise specified; PD-L1, programmed death-ligand 1.

^a^*t* test used for continuous and count variables, and χ^2^ test for binary and categorical variables, comparing patients tested vs. not tested for PD-L1.

^b^Reported percentages for race, smoking history, and stage pertain to patients with available data.

A total of 6024 (48%) patients had one or more tests for PD-L1 at any time from October 2, 2015, through December 31, 2017; the remaining 6550 (52%) patients in the study cohort were not tested for PD-L1 status. Patients whose NSCLC was tested for PD-L1 were more likely to have NSCLC initially diagnosed at stage IV (83% vs. 70% of those not tested; p < 0.001), of nonsquamous histology (76% vs. 68%; p < 0.001), and tested for *EGFR* mutation and *ALK* rearrangement (87% vs. 56%; p < 0.001; Table 1). Other statistically significant differences between patients who were tested for PD-L1 and those not tested appeared not to be clinically significant, for example, 36% vs. 33% of those with vs. without PD-L1 test, respectively, were age 65 years or younger.

### PD-L1 results by assay type

The 6024 patients had a total of 7031 PD-L1 tests, of which 4868 tests (69%) were included in the analyses, and 2163 tests (31%) were excluded because of missing assay type (n = 749), missing PD-L1 tumor expression data (n = 645), or missing assay type and results (n = 769). The 22C3 assay was the most common one used, comprising 78% of available assay results (Table 2). The LDTs, taken as a group, were the second most common assays used (15%), followed by the 28–8 assay (4%), and, least commonly, the SP142 assay (3%; Table 2).

**Table 2:** PD-L1 biomarker immunohistochemical (IHC) assay results by assay type^a^.

PD-L1 tumor expression, categorized	FDA-approved IHC assay			LDTs^c^ (n = 709)
	22C3 (n = 3799)	28–8 (n = 195)	SP142^b^ (n = 165)	
<1%	1469 (38.7)	76 (39.0)	96 (58.2)	260 (36.7)
1–49%	1085 (28.6)	57 (29.2)	52 (31.5)	289 (40.8)
≥50%	1245 (32.8)	62 (31.8)	17 (10.3)	160 (22.6)

Data presented as No. (%). Some patients had more than one test and are represented in more than one column. FDA, Food and Drug Administration; LDT, laboratory-developed test; PD-L1, programmed death-ligand 1.

^a^χ^2^ test: (1) p < 0.001 for comparing results across all 4 assay types: 22C3, 28–8, SP142, and LDTs (including the SP263); (2) p < 0.001 comparing results across three assay types (22C3, 28–8, and LDTs), excluding the SP142 assay; (3) p = 0.96 comparing results between 22C3 and 28–8.

^b^SP142 results represent the percentage of tumor cells staining for PD-L1 (immune cell staining not included).

^c^LDTs included 45 tests using the SP263, with 23 (51%), 13 (29%), and 9 (20%) showing PD-L1 tumor expression of <1%, 1–49%, and ≥50%, respectively.

Measured PD-L1 tumor expression, categorized as <1%, 1–49%, and ≥50%, varied significantly across the four assay types (χ^2^ p < 0.001) and across three assay types excluding the SP142 assay (χ^2^ p < 0.001). However, there was no significant difference between the 22C3 and 28–8 assays for PD-L1 tumor expression as categorized (χ^2^ p = 0.96; Table 2). A PD-L1 tumor expression of ≥50% was recorded approximately one-third of the time with the 22C3 and 28–8 assays, less frequently with LDTs (23%), and 10% of the time with the SP142. Of the 45 tests using the SP263 assay, 9 (20%) had recorded PD-L1 tumor expression ≥50% (results included with LDTs; see Table 2).

### PD-L1 testing trends

The percentage of patients whose NSCLC was tested for PD-L1 increased during the study period from 18% in the fourth quarter of 2015 to 71% in the third quarter of 2017 (Table 3). The 22C3 was the most common assay used in each quarter, with LDTs representing the second largest category. Assay type was not recorded for approximately 20% of tests in each quarter (Table 3).

**Table 3:** PD-L1 testing trends over time, by assay type.

Year and Quarter	No. Patients	Patients Tested, No. (% patients)	Number of PD-L1 Tests	PD-L1 IHC Assay Type,^a^ No. (% assays)					
				22C3 Assay	28–8 Assay	SP-142 Assay	SP-263 Assay	LDT	Unknown Assay Type
2015Q4	1511	275 (18.2)	326	127 (39.0)	17 (5.2)	3 (0.9)	2 (0.6)	91 (27.9)	86 (26.4)
2016Q1	1716	364 (21.2)	411	207 (50.4)	15 (3.6)	5 (1.2)	3 (0.7)	74 (18.0)	107 (26.0)
2016Q2	1532	384 (25.1)	443	239 (54.0)	22 (5.0)	8 (1.8)	1 (0.2)	68 (15.3)	105 (23.7)
2016Q3	1625	563 (34.6)	680	372 (54.7)	35 (5.1)	13 (1.9)	4 (0.6)	106 (15.6)	150 (22.1)
2016Q4	1425	881 (61.8)	1066	579 (54.3)	37 (3.5)	55 (5.2)	7 (0.7)	149 (14.0)	239 (22.4)
2017Q1	1435	1049 (73.1)	1247	773 (62.0)	40 (3.2)	51 (4.1)	10 (0.8)	141 (11.3)	232 (18.6)
2017Q2	1365	1043 (76.4)	1197	791 (66.1)	28 (2.3)	18 (1.5)	8 (0.7)	97 (8.1)	255 (21.3)
2017Q3	1229	940 (76.5)	1071	686 (64.1)	21 (2.0)	16 (1.5)	19 (1.8)	97 (9.1)	232 (21.7)
2017Q4	736	525 (71.3)	590	402 (68.1)	9 (1.5)	3 (0.5)	11 (1.9)	53 (9.0)	112 (19.0)
Total	**N = 12,574**	**N = 6024 (47.9)**	**N = 7031**	**N = 4176 (59.4)**	**N = 224 (3.2)**	**N = 172 (2.4)**	**N = 65 (0.9)**	**N = 876 (12.5)**	**N = 1518 (21.6)**

IHC, immunohistochemical; LDT, laboratory-developed test; PD-L1, programmed death-ligand 1; Q, quarter.

^a^Some patients had more than one test and/or type of assay and could be represented in more than one assay type column.

## Discussion

We found no significant difference in measured PD-L1 tumor expression between the 22C3, the most common assay type, and the 28–8 assay, in this observational study of PD-L1 testing patterns for patients with metastatic or recurrent NSCLC at US oncology practices. These findings indicate that the 22C3 and 28–8 can be used interchangeably for measuring PD-L1 tumor expression in NSCLC. Most importantly, however, the results of LDTs and particularly the SP142 assay appeared discordant, with proportionately fewer PD-L1 tumor expression results of ≥50%, suggesting that some cases of NSCLC with high PD-L1 tumor expression could have been missed.

Recent study results have indicated that pembrolizumab administered in the first-line setting may be more effective than in the second-line or later setting, as demonstrated in the KEYNOTE-024 trial and a recent large meta-analysis [3,20]. Therefore, in the clinical setting, correctly identifying patients with NSCLC PD-L1 tumor expression score ≥50% and thus eligible for first-line therapy with pembrolizumab is imperative.

Our study evaluated concordance of PD-L1 assays in real-world oncology practice where most patients are treated. This enabled us to include almost 5000 NSCLC samples, a much higher number than those examined in prior quality-controlled, laboratory-based comparative studies (from 21 to 493 samples) [6,10,11,15–19,23,24]. Our findings support the results of prior work, including the Blueprint study phases 1 and 2, which found that the SP142 consistently underperforms as compared with the 22C3 and the 28–8 [10,15].

We included the SP263 in the LDT category because the SP263 was not approved for use in NSCLC in the US during the study. Only 45 tests used the SP263, too few from which to draw conclusions. In Blueprint phases 1 and 2 [10,15], as well as in other prior studies [11,16–18], the results of the SP263 were considered to be aligned with those of the 22C3 and/or the 28–8, while in two studies, the SP263 stained more tumor cells on some samples relative to the other assays [19,24].

We found that LDTs constituted a substantial proportion of all PD-L1 assays (15%) included in the database. The overall percentage of tumors with measurable PD-L1 tumor expression (≥1%) using LDTs (63%) was similar to that using the 22C3 (61%) and the 28–8 (61%); however, the proportion with PD-L1 ≥50% was much lower (23% vs. 33% and 32%, respectively), suggesting that the LDTs as a group could be less sensitive IHC assays. Two recent studies examining interlaboratory and interassay concordance both found that locally developed LDTs produced variable results, some aligned and some not aligned with those of the 22C3, 28–8, and SP263 [11,18]. Other studies report good concordance among the 22C3 and 28–8 and specific validated LDTs [23,25]. These results together with ours suggest that all LDTs should be carefully validated and that clinicians should exercise caution in relying on LDTs for PD-L1 IHC assays.

The implementation of NSCLC testing recommendations in the clinical setting can be challenging, particularly as NSCLC guideline recommendations have expanded to testing for molecular biomarkers other than *EGFR/ALK* genomic alterations, including *ROS1* and *BRAF*, in addition to PD-L1 [26,27]. Primary lung tumors often have low tumor cellularity, and obtaining adequate tissue quantity and quality for both histological subtyping of NSCLC as well as molecular and biomarker testing may be difficult [27].

Strengths of this observational study include the large sample size, the very recent, up-to-date findings, and the use of a large, well-maintained database drawing on longitudinal medical records from a geographically diverse patient population in the US [21]. Our results reflect current clinical practice in the US, with PD-L1 readings by numerous different pathologists. Not surprisingly, we found that the percentage of patients whose NSCLC was tested for PD-L1 increased substantially from the fourth quarter of 2015 through the fourth quarter of 2017.

Several limitations of this study should be considered in interpreting our analyses. The Flatiron data primarily reflect real-world clinical practices at community oncology centers (~90% of patients in this study), with fewer patients seen or beginning treatment at academic medical centers, for example. We lacked some information that would have been useful to assess the generalizability of our findings, including the types of testing laboratories, handling of diagnostic material, and the specimens themselves—whether histologic or cytologic, and whether newly obtained or archived specimens. Moreover, data were missing regarding assay type or results for 31% of the recorded tests. Finally, the study dataset did not include response data associated with the PD-L1 test results.

Continuing follow-up over time with more complete characterization of PD-L1 test specimens will be important to supplement our findings. The rapid increase in rates of PD-L1 testing, as observed in this study, will facilitate future study of the impact of PD-L1 assay type on the reported level of PD-L1 tumor expression. In addition, information about patient outcomes associated with PD-L1 tumor expression levels and chosen therapies in the real-world setting is of great interest. Other areas for further research include identifying and addressing barriers to PD-L1 testing in clinical practice.

In conclusion, our findings suggest that the 22C3 and 28–8 assays can be used interchangeably for measuring PD-L1 tumor expression in metastatic or recurrent NSCLC. Instead, use of the SP142 assay or an LDT for IHC PD-L1 screening is associated with less reliable capture of PD-L1 positivity, particularly at the higher cut-point of ≥50% PD-L1 tumor expression. This could result in a missed opportunity for first-line monotherapy with an anti-PD-1/anti-PD-L1 agent and optimizing outcomes for patients with metastatic NSCLC.
